# Anti‐inflammatory effects of kawakawa (*Piper excelsum*): An integrative mRNA–miRNA approach

**DOI:** 10.1002/fsn3.4450

**Published:** 2024-09-10

**Authors:** Senilaite Tautuiaki, Jerusha Gojer, Ramya Jayaprakash, Pankaja Sharma, Chris Pook, Meika Foster, Jennifer Miles‐Chan, Richard Mithen, Farha Ramzan

**Affiliations:** ^1^ Liggins Institute The University of Auckland Auckland New Zealand; ^2^ School of Biological Sciences The University of Auckland Auckland New Zealand; ^3^ Edible Research Ltd Christchurch New Zealand; ^4^ AuOra Ltd, Wakatū Incorporation Nelson New Zealand

**Keywords:** anti‐inflammatory diet, gene expression, inflammation, microRNA, PBMCs, tea

## Abstract

Kawakawa (*Piper excelsum*) is an endemic medicinal plant widely consumed by Māori in New Zealand. Presence of diverse biologically active phytochemicals in kawakawa may underpin its putative therapeutic anti‐inflammatory properties. However, no human studies on its anti‐inflammatory effects are yet undertaken. Blood samples from a randomized controlled dietary intervention exploring the impact of kawakawa compared to control on postprandial microRNAs (miRNA) abundances and their respective gene and protein targets in a cohort of healthy human volunteers (*n* = 26; Age; 33.6 ± 1.9 year and BMI; 22.5 ± 0.4 kg/m^2^) were analyzed. Postprandial levels of nine miRNAs showed differential abundances; hsa‐miR‐17‐5p, ‐21‐5p, ‐320a‐5p, let‐7g‐5p, ‐16‐5p, ‐122‐5p, and ‐144‐3p was upregulated while as hsa‐miR‐221‐3p and ‐223‐3p was downregulated in response to kawakawa compared to control. In silico analysis indicated enrichment of miRNAs in multiple inflammation‐related pathways, including apoptosis, cytokine signaling, MAPK signaling, and MTOR pathways. Furthermore, gene expression of *IL‐8* (*p* = .03), *IL‐6* (*p* = .01), and *PPAR‐γ* were significantly reduced following kawakawa intake compared to control. While as plasma IL‐6 showed a significant increase over 120 min in the kawakawa arm. These results highlight kawakawa to exert anti‐inflammatory effects by modulating the expression of miRNAs and their target genes and proteins in the inflammatory signaling pathways.

## Graphical abstract

Acute intake of kawakawa tea, derived from the indigenous New Zealand plant *Piper excelsum*, demonstrates promising anti‐inflammatory effects. This effect is mediated through the regulation of miRNA expression, which in turn influences their target genes and proteins associated with key inflammatory signaling pathways. The modulation of these pathways suggests that kawakawa tea could play a significant role in reducing inflammation by offering a natural and sustainable dietary intervention for managing inflammation‐related health conditions.

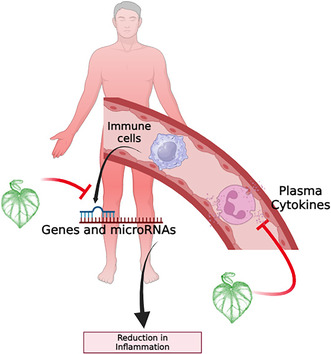

## 1 INTRODUCTION

Kawakawa (*Piper excelsum* G. Forst) is a native plant of New Zealand that holds both cultural and medicinal (Rongoā Māori) importance for Māori, the indigenous people (Briggs, 1941; Koia & Shepherd, 2020). The leaves of the kawakawa plant are shown to contain diverse biologically active phytochemicals, including but not limited to lignans (excelsin and diayangambin); amides (piperine, pellitorine, dihydropiperlonguminine, and fagaramide); phenylpropanoids (elemicin and myristicin); flavone glycoside (vitexin); and terpenoids (α‐pinene) (Jayaprakash et al., 2022). In vitro and in vivo studies exploring the biological properties of these chemicals from a range of different plant sources have reported numerous potential health effects, including anti‐diabetic and anti‐inflammatory properties (Koia & Shepherd, 2020); their presence also in kawakawa could contribute to its therapeutic uses in rongoā Māori.

Interestingly, the bioactive compounds such as myristicin, elemicin, piperine, and isovetexin identified in kawakawa are shown to possess anti‐inflammatory properties. For example, Myristicin is shown to inhibit the production of several pro‐inflammatory cytokines such as NO, IL‐6, and IL‐10 in mouse macrophages and THP‐1 monocytes (Lee & Park 2011). Piperine also found in black pepper is also reported to inhibit the production of IL‐2 (Končarević et al., 2014), a pro‐inflammatory cytokine released from Th‐1 cells and is involved in the activation of T cells and production of other pro‐inflammatory molecules. Isovetexin, a glycosyl flavone is also reported to show anti‐inflammatory effects by reducing the production of TNF‐α (Ramzan, D'Souza, Durainayagam, Milan, Roy, et al., 2020) in immune cell models.

While there is existing evidence of the bioactive effects of kawakawa in animal and cell models (Butts et al., 2019; Lei et al., 2015), limited research on its effects in humans exist. For the first time, we have shown the effects of kawakawa intake by healthy human volunteers on glycemic response (Ramzan et al., 2022). However, how kawakawa exerts these effects on humans remains uncertain.

microRNAs (miRNAs) are small noncoding‐RNAs with widespread biological functions (O'Brien et al., 2018), acting as negative regulators of post‐transcriptional gene expression (Shuai Jiang, 2016). Circulatory miRNAs are known to play a critical role in cell‐to‐cell communication (Fatima & Nawaz, 2017) and have been increasingly identified as potential biomarkers of disease states, prognosis, and the progression of different conditions including type 2 diabetes mellitus (T2DM) and cardiovascular disease (CVD) (Max et al., 2018; Ramzan, D'Souza, Durainayagam, Milan, Markworth, et al., 2020). Evidence demonstrates miRNAs to regulate expression of a wide range of genes and proteins involved in metabolic pathways (Åkerman et al., 2018; Gong et al., 2018) including the insulin signaling and inflammatory pathways.

Based on the functional role of miRNAs, we hypothesize that acute intake of kawakawa would result in differential abundance of miRNAs related to glycemic response and inflammatory pathways. Furthermore, this altered miRNA abundance would correspond with the dysregulated expression of genes and proteins with known metabolic regulatory functions. Therefore, the current study aimed to quantify the abundance of circulatory miRNAs in response to an acute intake of kawakawa tea. Based on in silico functional target analysis of the differentially regulated miRNAs, target genes of these miRNAs were isolated and analyzed from the peripheral blood mononuclear cells (PBMCs).

## 2 METHODS

### 2.1 Study design and population

The plasma samples utilized in this study were archived from the previously conducted dietary intervention (TOAST study), approved by the Health and Disabilities Ethics Committee (HDEC) Auckland, New Zealand (20/STH/236) (Ramzan et al., 2022). The study was conducted in accordance with the Declaration of Helsinki guidelines. All participants provided written informed consent. The study was prospectively registered with the Australian New Zealand Clinical Trials Registry at www.anzctr.org.au: ACTRN12621000311853.

A total of 30 participants were recruited; however, one withdrew after the initial intervention, and three were excluded due to elevated fasting glucose levels. Therefore, samples from only 26 participants (11 males and 15 females) were obtained for this analysis.

### 2.2 Interventions and sample collection

**FIGURE 1:**
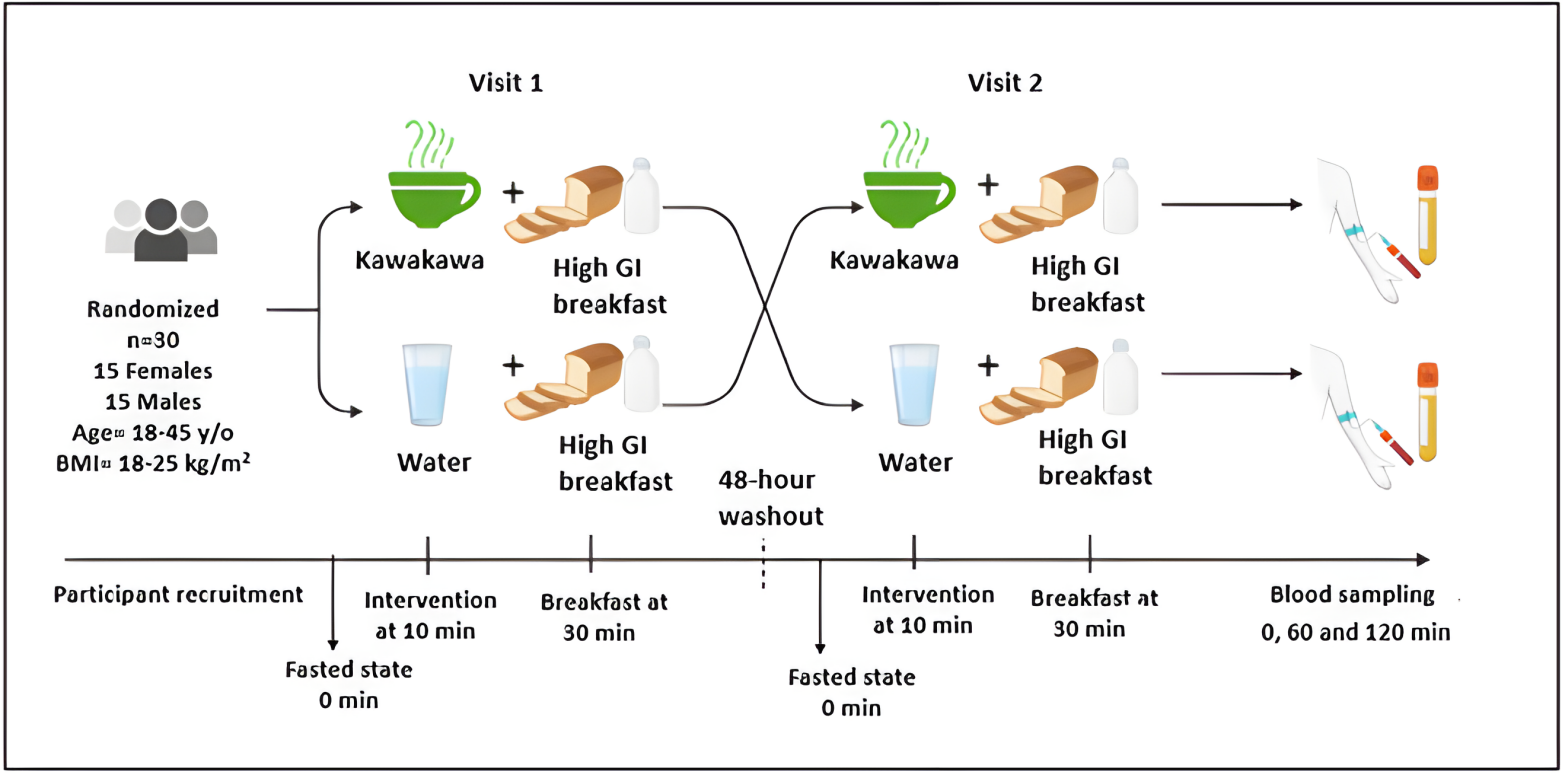
Overview of TOAST study design. BMI, body mass index; GI, glycemic index; min, minutes; *n*, number; y/o, year old.

Participants were provided with a standardized dinner prior to the intervention day and were asked to fast overnight (10–12 h) and arrive at the Liggins Institute, Clinical Research Unit, between 7:00 a.m. and 8:00 a.m. on each of the two intervention days. At visit 1, they were assigned to one of two interventions (Figure 1) in a randomized sequence with a 48h gap between each intervention.

The interventions consisted of consuming, after a 10‐min fasting baseline measurement, either: (i) 250 mL of kawakawa tea (made by infusing 4 g of dried kawakawa leaves from ŌKU Ltd, New Zealand, in 250 mL of 70–80°C hot water for 10 min); or (ii) drinking only 250 mL of hot water (70–80°C).

After consuming the tea or hot water, participants were given a 10 min window (at the 30 min mark) to consume a high‐carbohydrate breakfast comprising two slices of white bread (935 kJ), 10 g of strawberry jam (80 kJ), and 250 mL of rice milk (578 kJ). Fasting (0 min) and postprandial (60 and 120 min) venous blood samples were collected at each visit, as described below.

### 2.3 Sample collection

Baseline fasting (time = 0 min) and postprandial (time = 60 and 120 min) venous blood samples were collected in EDTA‐coated vacutainers from each participant at each visit and immediately stored on ice before processing. Plasma was separated and aliquoted within 120 min of collection by centrifugation at 1900 ***g*** for 15 min at 4°C and was then immediately stored at −80°C until further analysis. PBMCs were extracted from whole blood samples following the Ficoll‐Paque method of extraction (Ramzan, D'Souza, Durainayagam, Milan, Roy, et al., 2020).

### 2.4 Biochemical analysis

Baseline fasting and postprandial glucose (nmol/L) analysis was performed on the Hitachi 902 autoanalyzer (Hitachi High Technologies Corporation, Japan) using an enzymatic colorimetric assay (Sepulveda, 2019). Fasting plasma and postprandial insulin (mU/L) and triglycerides (mmol/L) were measured using a Cobas Modular P800 autoanalyzer using the microparticle enzyme immunoassay (Roche Diagnostics, Auckland, New Zealand). The area under the curve (AUC) of plasma glucose and insulin concentration was calculated using the trapezoid method. The Homeostatic Model Assessment for Insulin Resistance (HOMA‐IR), an index used to determine if a patient has insulin resistance (Salgado et al., 2010), was subsequently calculated according to the following equation:

$$\text{HOMA}‐\mathrm{IR}=\text{fasting insulin}\;\left(\mathrm{mU}/L\right)\times \text{fasting glucose}\;\left(\text{nmol}/L\right)/22.5$$

### 2.5 Plasma and PBMC RNA isolation

250 μL plasma was used for total RNA extraction (including miRNAs) using a previously described protocol by Ramzan, D'Souza, Durainayagam, Milan, Roy, et al. (2020). A fixed plasma volume was utilized to minimize extraction variation between different samples and time points (El‐Khoury et al., 2016).

Total RNA was isolated from approximately 2.5 × 10^6^ PBMCs collected at each time point using the AllPrep® DNA/RNA/miRNA Universal Kit (QIAGEN, Germany), following the manufacturer's protocol (Ramzan et al., 2019).

### 2.6 cDNA synthesis and circulating miRNA quantitative PCR (qPCR)

A fixed volume of 2 μL of total RNA was used as an input for the cDNA synthesis reaction using TaqMan™ Advanced miRNA cDNA Synthesis Kit (Catalogue number: A28007, Applied Biosystems, USA), according to the manufacturer's recommendations. For quantification of plasma miRNA abundances using qPCR analysis, custom human miRNA assays of hsa‐miR‐15a‐5p, ‐16‐5p, ‐17‐5p, ‐21‐3p, ‐21‐5p,‐122‐5p, ‐144‐5p, ‐320a, ‐221‐3p, ‐222‐3p, ‐223‐3p, and let‐7g‐5p were used (TaqMan MicroRNA Assays, Applied Biosystems, USA). Quantification was performed on a Quant Studio™ 12 Flex Real‐Time PCR System (Thermo Fisher Scientific, USA), and the detected cycle threshold (*C*_t_) of ≤35 was set for the miRNA analysis.

For the normalization of abundant data, a geometric mean of three endogenous miRNAs (hsa‐miR‐423‐5p (Damanti et al., 2021; Wang et al., 2016), hsa‐miR‐451a‐5p (Elshelmani et al., 2021), and hsa‐miR‐191‐5p (Damanti et al., 2021; Wang et al., 2016)) was used (Vandesompele et al., 2002a). Hemolysis of all samples was monitored by comparing hsa‐miR‐451a abundance (a highly expressed miRNA in red blood cells) with hsa‐miR‐23a‐3p abundance (a miRNA unaffected by hemolysis) (Shah et al., 2016). The resulting Δ*C*_t_ (miR‐23a‐3p‐miR‐451a) was used to measure the degree of hemolysis; samples with an Δ*C*_t_ of >7 were excluded from further analysis. The abundance of miRNAs was measured using the 2 (^−ΔΔCt^) method (Schmittgen & Livak, 2008).

### 2.7 In silico target analysis

A miRNA‐target gene network was built using miRNet (https://www.mirnet.ca/) by including the differentially abundant miRNAs (Fan et al., 2016). All sets of genes targeted by the miRNAs were identified and were subsequently used for the prediction of pathways targeted by these miRNAs. Functional annotation of the dysregulated miRNAs and the identification of miRNA‐target gene‐controlled pathways were determined via the Kyoto Encyclopedia of Genes and Genomes (KEGG) pathway based on the hypergeometric tests with *p* values ≤.05 adjusted for false discovery rate (FDR).

### 2.8 qPCR gene expression analysis

Input RNA of 350 μg was used for cDNA synthesis using the SuperScript IV VILO Master Mix (Thermo Fisher USA). Quantification of gene expression (mRNA) was performed by qPCR on a LightCycler 480 II (Roche Applied Science, Germany) using TaqMan Universal Master Mix II (Thermo Fisher, USA). Genes quantified (Table 1) included peroxisome proliferator‐activated receptor gamma (*PPAR*‐*γ*), Fas Cell Surface Death Receptor (*FAS*), C‐reactive protein (*CRP*), cluster of differentiation 36 (*CD36*) and AMP‐activated kinase (*AMPK*) and pro‐inflammatory cytokines (tumor necrosis factor‐alpha (*TNF‐α*), interleukin‐2 (*IL‐2*), interleukin‐6 (*IL‐6*), and interleukin‐8 (*IL‐8*)). Primers for qPCR were designed using BLAST software (Ye et al., 2012). For normalization of the PCR data, the geometric mean (Vandesompele et al., 2002b) of three human reference genes (Eisenberg & Levanon, 2013; van de Moosdijk & van Amerongen, 2016), charged multivesicular body protein 2A (*CHMP2A*), valosin‐containing protein (*VCP*), and chromosome 1 open reading frame 43 (*C1orf43*), was used. The relative gene expression was measured using the 2(^ΔCt^) method (Schmittgen & Livak, 2008).

**TABLE 1:** Forward and reverse primer nucleotide sequences (5′–3′) for target genes, primer length, GC%, and melting temperatures of each target primer.

Target primer	Primer sequence (5′–3′)	Length	GC%	*T*_m_
*IL‐2*	Forward: CTCAAACCTCTGGAGGAAGTGC	22	54.5	60.8
	Reverse: CAATGGTTGCTGTCTCATCAG	22	50.0	60.1
*IL‐6*	Forward: GCCCACCGGGAACGAAAGAGA	21	61.9	64.6
	Reverse: ACCGAAGGCGCTTGTGGAGA	20	60.0	64.2
*IL‐8*	Forward: AGAGCTCTGTCTGGACCCCA	20	60.0	62.1
	Reverse: TTCTCAGCCCTCTTCAAAAACTTC	24	41.6	59.4
*FAS*	Forward: GTTGGTGGACCCGCTCAGTA	20	60.0	61.8
	Reverse: TAGGAGGGTCCAGATGCCCA	20	60.0	61.9
*TNF‐α*	Forward: AACCCTCAGACGCCACATCC	20	60.0	62.1
	Reverse: TGGAGCCGTGGGTCAGTATG	20	60.0	61.6
*PPAR‐γ*	Forward: TGGTGACCAGAAGCCTGCAT	20	55.0	61.7
	Reverse: CCCAAAGTTGGTGGGCCAGA	20	60.0	62.6
*AMPK*	Forward: ATCATCACCTGACTCGGCCC	20	60.0	61.6
	Reverse: TATGGCGTGCCCTTGGTGTT	20	55.0	62.4

Abbreviations: AMPK, AMP‐activated kinase; FAS, FAS cell surface death receptor; GC%, guanine‐cytosine percentage; IL‐2, interleukin‐2; IL‐6, interleukin‐6; IL‐8, interleukin‐8; PPAR‐γ, peroxisome proliferator activated receptor‐gamma; *T*_m_, melting temperature; TNF‐α, tumor necrosis factor‐alpha.

### 2.9 Protein quantification: Enzyme‐linked immunoassay (ELISA)

An immunoassay procedure was conducted to analyze the protein concentrations of plasma inflammatory cytokines, including IL‐1β, IL‐2, IL‐6, IL‐8, and TNF‐α, using the MILLIPLEX Human Cytokine/ Chemokine/Growth Factor Panel A Magnetic Bead Panel kit.

### 2.10 Statistical analysis

The expression data were evaluated for normality using the Shapiro–Wilk test. The differences in the abundance of plasma miRNA, PBMC genes, AUC _glucose_, AUC _insulin_, and AUC_TG_ in relation to the acute dose of the intervention were measured using a mixed effects model with time as a repeated factor and group as a between‐subject factor, followed by Holm–Sidak multiple comparison corrections. Samples with an expression of more than three times the interquartile range were treated as outliers and were subsequently removed from further analysis (Arenas et al., 2016). Data are shown as means ± SEM unless otherwise stated. Analyses were conducted using SPSS version 25.0 (SPSS Inc., USA), and graphs were constructed using GraphPad prism‐9 (GraphPad Software, USA). Statistical significance was set at *p* ≤ .05.

## 3 RESULTS

### 3.1 Study population characteristics

The baseline characteristics of the study participants are summarized in Table 2. The study population included 26 self‐reported healthy participants; male (*n* = 11) and female (*n* = 15), aged 18–45 years and BMI range of 18–25 kg/m^2^. The male and female participants differed in body weight (*p* = .003) but not in BMI.

**TABLE 2:** Baseline clinical and demographic characteristics of the study participants.

Variables	Male	Female	*p*‐Value	All
Number of participants	11	15		26
Age (years)	34.2 ± 2.0	32.3 ± 1.4	.55	33.6 ± 1.9
Body weight (kg)	66.5 ± 2.0	58.8 ± 1.8	**<.01**	67.9 ± 2.0
BMI (kg/m^2^)	22.1 ± 0.4	22.3 ± 0.4	.63	22.5 ± 0.4
Glucose (mmol/L)	5.1 ± 0.07	4.9 ± 0.1	.16	6.1 ± 0.1
Triglycerides (mmol/L)	0.9 ± 0.1	0.9 ± 0.1	.70	0.9 ± 0.04
Insulin (mU/L)	47.9 ± 6.2	55.6 ± 6.0	.47	52.5 ± 4.3
HOMA‐IR	16.1 ± 2.4	17.8 ± 2.4	.38	17.3 ± 1.7

*Note*: Data presented as mean ± SEM. Significance level *p* ≤ .05 (highlighted in bold). The stated *p*‐value is from comparing males and females (average baseline of both visits) using a one‐way ANOVA test.

Abbreviations: ANOVA, analysis of variance; BMI, body mass index; BP, blood pressure; HOMA‐IR, homeostasis model assessment‐estimated insulin resistance.

### 3.2 Baseline abundance of targeted circulatory miRNAs

The circulatory abundance of targeted miRNAs and the endogenous controls at baseline are summarized in Table 3. The baseline abundance did not differ between the two study interventions (kawakawa tea and water).

**TABLE 3:** Baseline abundance of targeted circulatory miRNAs and endogenous controls between study interventions.

microRNA	Kawakawa	Water	*p*‐Value
hsa‐miR‐15a‐5p	0.62 ± 0.08	0.45 ± 0.05	.07
hsa‐miR‐16‐5p	2.27 ± 0.50	2.24 ± 0.46	.95
hsa‐miR‐17‐5p	1.72 ± 0.41	1.00 ± 0.19	.11
hsa‐miR‐21‐3p	0.02 ± 0.01	0.01 ± 0.00	.24
hsa‐miR‐21‐5p	0.01 ± 0.00	0.04 ± 0.00	.10
hsa‐miR‐122‐5p	0.04 ± 0.01	0.07 ± 0.01	.13
hsa‐miR‐144‐3p	1.11 ± 0.28	1.12 ± 0.17	.96
hsa‐miR‐221‐3p	0.68 ± 0.18	0.36 ± 0.10	.13
hsa‐miR‐223‐3p	2.02 ± 0.56	1.68 ± 0.46	.64
hsa‐miR‐222‐3p	0.08 ± 0.03	0.06 ± 0.01	.39
hsa‐miR‐320a	0.88 ± 0.18	0.84 ± 0.08	.25
let‐7g‐5p	0.13 ± 0.03	0.12 ± 0.01	.22
Endogenous control			
hsa‐miR‐451a‐5p	15.9 ± 14.9	13.1 ± 15.2	.11
hsa‐miR‐191‐5p	0.5 ± 0.08	0.5 ± 0.1	.18
hsa‐miR‐423‐5p	0.5 ± 0.06	0.4 ± 0.05	.14

*Note*: Data presented as mean ± SEM. Significance level ≤.05. The stated *p*‐value is from comparing study interventions at baseline using a one‐way ANOVA test. K, kawakawa; W, water; ANOVA, analysis of variance.

### 3.3 Postprandial abundance of circulatory miRNAs

**FIGURE 2:**
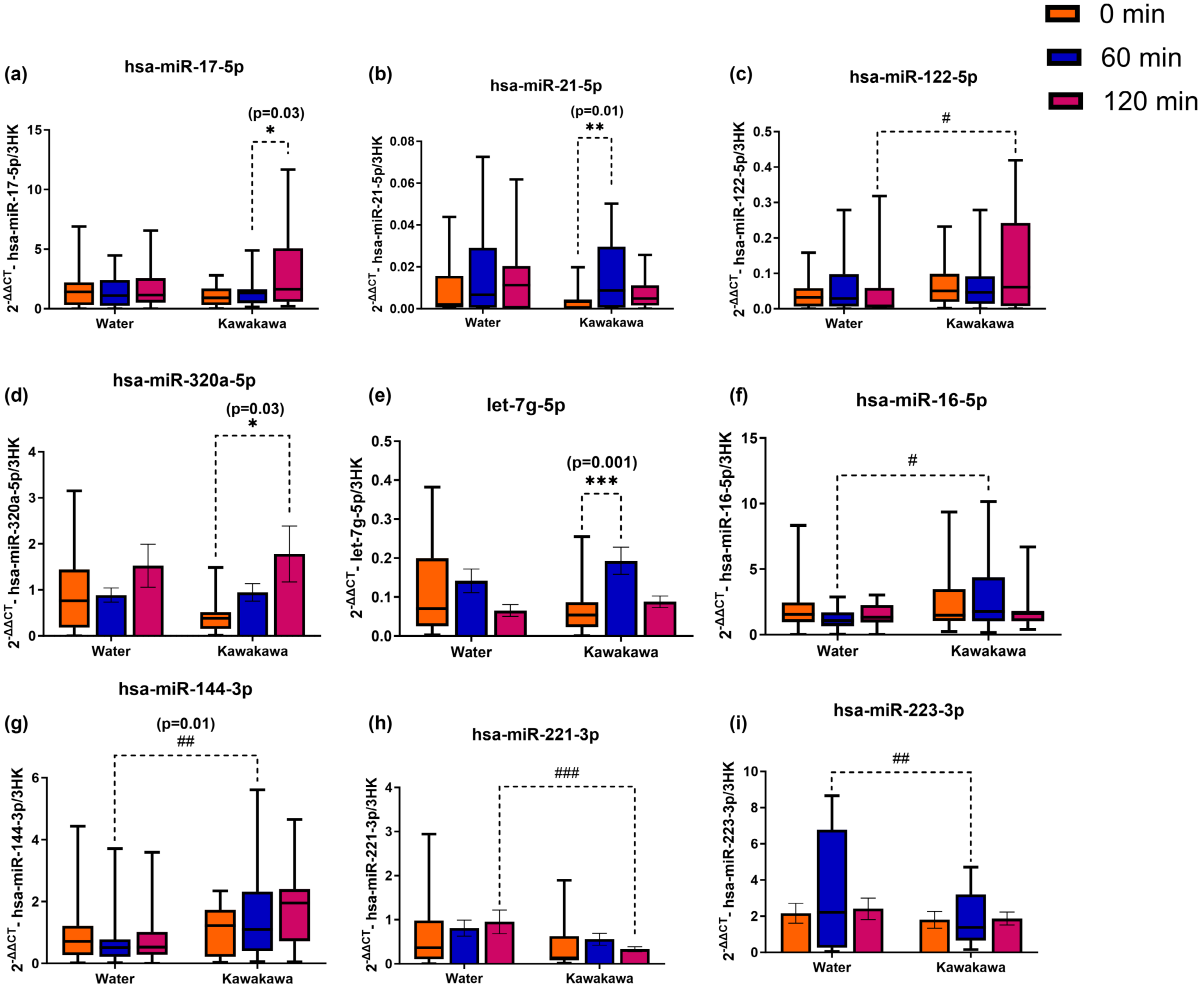
Postprandial abundance of circulatory miRNAs. Different colors represent time points at fasting (0 min, orange color), at 60 min (Blue color) and at 120 min (Red color); *denotes a significant difference within the groups: *p ≤ .05, **p ≤ .01, ***p ≤ .001; ^#^denotes a significant difference between the groups: ^#^ *p* ≤ .05, ^##^ *p* ≤ .01, ^###^ *p* ≤ .001.

The postprandial abundance of circulatory miRNAs following kawakawa tea differed from the water control. Of the 12 analyzed, nine miRNAs showed differential abundances following the two interventions (Figure 2). A time‐point (0, 60, and 120 min) effect was observed in hsa‐miR‐17‐5p (*p* = .03), hsa‐miR‐21‐5p (*p* = .01), hsa‐miR‐320a‐5p (*p* = .03) and let‐7g‐5p (*p* = .001). An intervention (kawakawa and water) effect was observed in hsa‐miR‐16‐5p (*p* = .03), hsa‐miR‐122‐5p (*p* = .01), hsa‐miR‐144‐3p (*p* = .01), hsa‐miR‐221–3p (*p* < .01), and hsa‐miR‐223‐3p (*p* = .01). The abundance of hsa‐miR‐17‐5p, hsa‐miR‐21‐5p, hsa‐miR‐320a‐5p, let‐7g‐5p, hsa‐miR‐16‐5p, hsa‐miR‐122‐5p, and hsa‐miR‐144‐3p was upregulated in response to kawakawa intake compared to water, while as abundance of hsa‐miR‐221‐3p and hsa‐miR‐223‐3p downregulated on kawakawa intake compared to water. The abundance of hsa‐miR‐15a‐5p, hsa‐miR‐222‐3p, and hsa‐miR‐21‐3p remained unaltered in response to either of the interventions and were excluded from further analysis.

### 3.4 Correlation analysis

**FIGURE 3:**
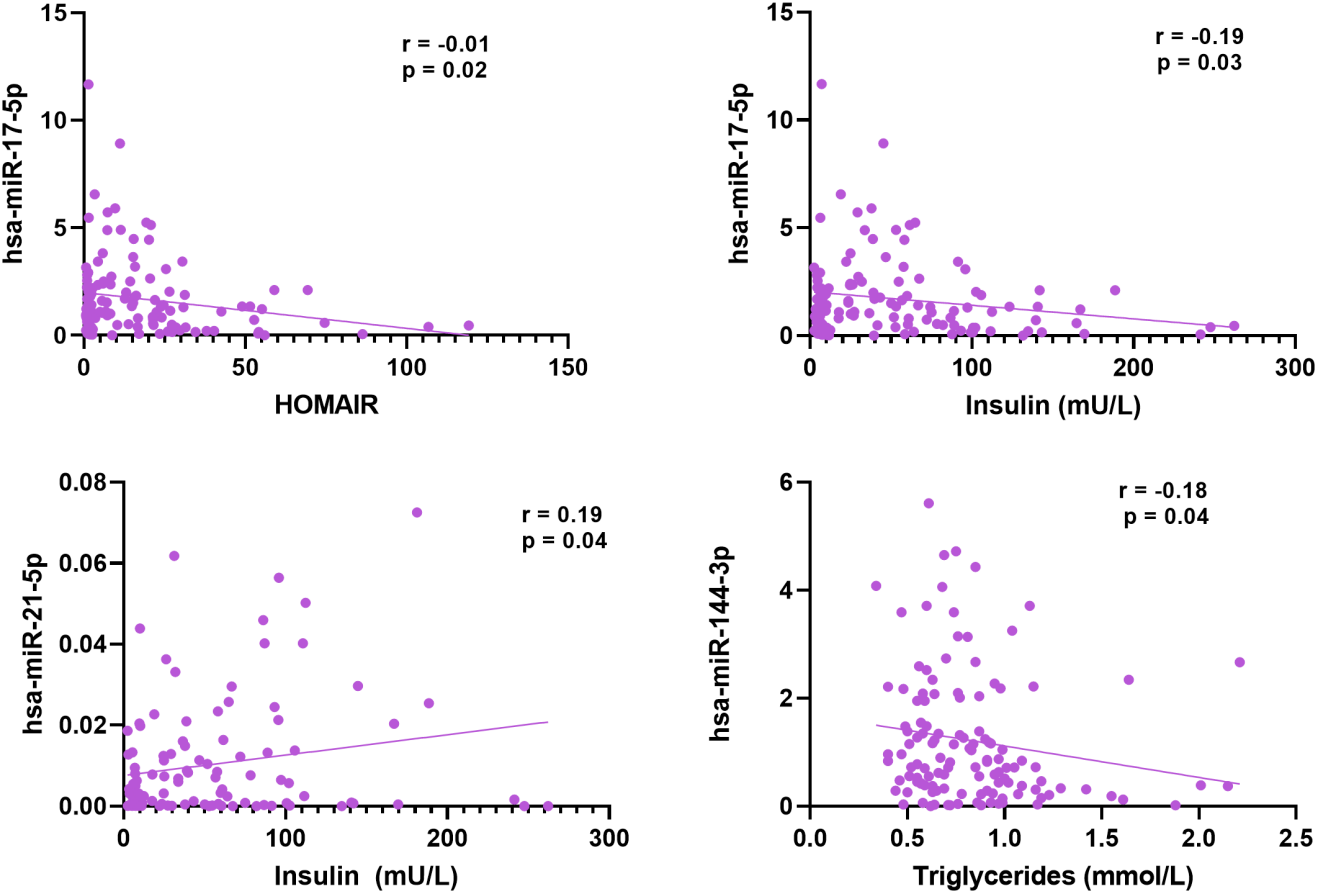
Pearson's correlation plots. The biochemical parameters are plotted on the *X*‐axis, and the relative microRNA expression ($2^{-∆∆C_{t}}$) is on the *y*‐axis. *r*‐correlation coefficient; *p*‐significance value.

A Pearson's correlation analysis was performed to establish the relationships between the differentially abundant miRNAs and biochemical biomarkers of metabolic health (Figure 3). There was a weak, negative correlation between hsa‐miR‐17‐5p with HOMA‐IR and plasma insulin (*r* = −.01, *p* = .02 and *r* = −.18, *p* = .03, respectively). A weak, positive correlation was observed between hsa‐miR‐21‐5p and plasma insulin (*r* = .10, *p* = .04), and hsa‐miR‐144‐3p showed a weak, negative correlation with plasma triglycerides (*r* = −.18, *p* = .04). However, no statistically significant correlation was observed between glucose and abundance of the other altered miRNAs.

### 3.5 Prediction of downstream mRNAs

**FIGURE 4:**
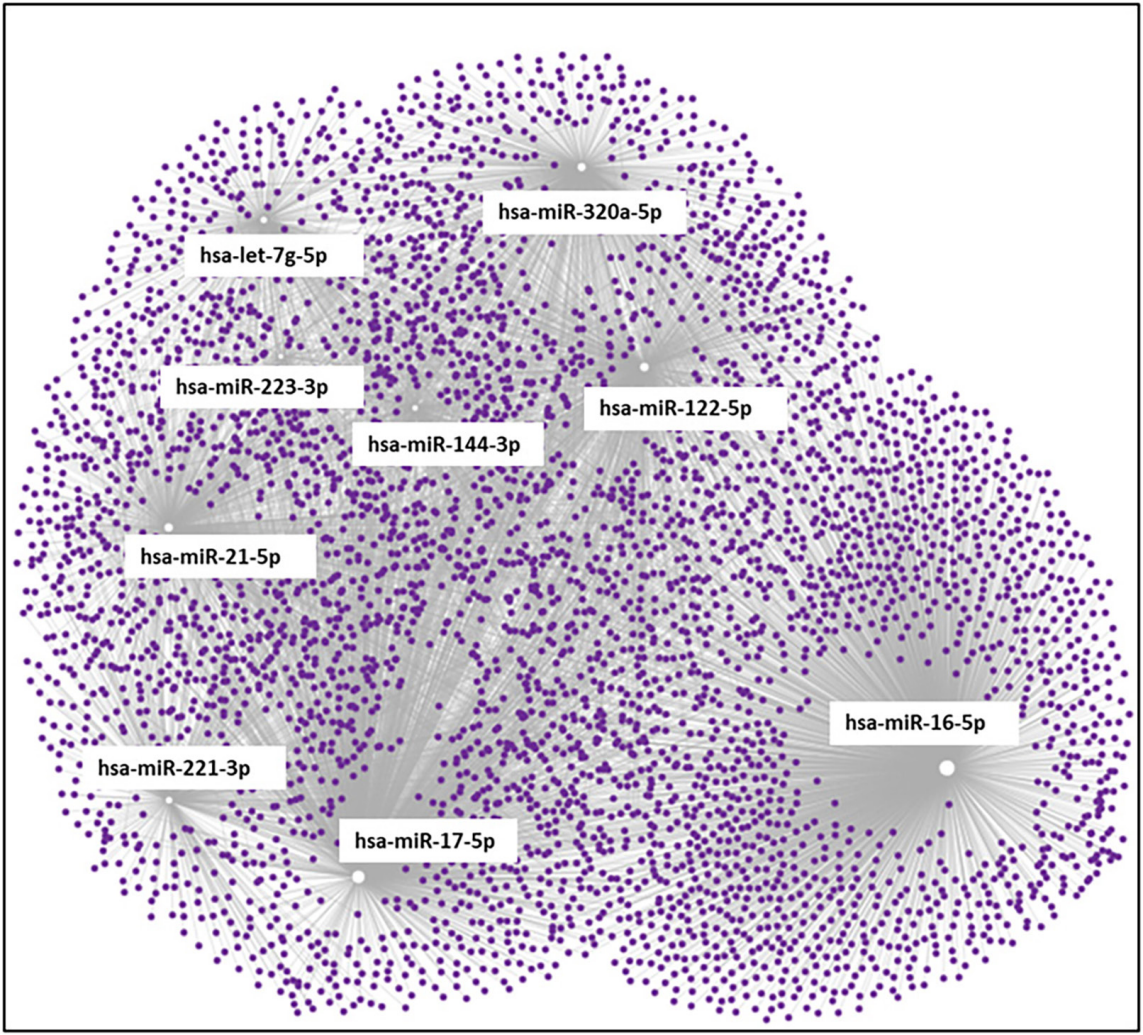
Functional enrichment analysis. Network visualization of the significant circulatory miRNA (White boxes) and their respective gene targets (purple dots).

Target gene prediction analysis demonstrated 4277 genes (both strong and weak interactions) as being putatively regulated by the differentially abundant miRNAs; hsa‐miR‐16‐5p, ‐17‐5p, ‐21‐5p, ‐122‐5p, ‐144‐3p, ‐221‐3p, ‐223‐3p, ‐320a‐5p, and let‐7g‐ 5p (Figure 4). The magnitude of the genes targeted by a single miRNA is proportional to the node size. For example, hsa‐miR‐16‐5p and hsa‐miR‐17‐5p target and share the highest number of genes within the network; hsa‐miR‐16‐5p alone targets approximately 1557 genes; and hsa‐miR‐17‐5p targets 1181 genes, as shown in Table 4.

**TABLE 4:** List of microRNAs and the total number of genes targeted individually.

microRNA	Total number of target genes
hsa‐mir‐16‐5p	1557
hsa‐mir‐17‐5p	1181
hsa‐mir‐21‐5p	612
hsa‐mir‐221‐3p	368
hsa‐mir‐223‐3p	98
let‐7g‐5p	341
hsa‐mir‐122‐5p	610
hsa‐mir‐144‐3p	211
hsa‐mir‐320a	584

The hypergeometric algorithm‐based functional enrichment analysis, with a false discovery rate (FDR) of *p* ≤ .05, identified that the gene targets of the nine differentially abundant miRNAs are associated with several inflammatory and related pathways, including apoptosis, cytokine signaling, MAPK signaling, and MTOR pathways. In particular, these miRNAs were shown to target the genes of inflammatory cytokines, including TNF‐α, FAS, IL‐1β, 1 L‐1α, and TGF‐β1. Modifications in these pathways have been previously described as associated with regulating metabolic homeostasis (Kaplon et al., 2022; Metallo & Vander Heiden, 2013; Slack, 2017). Additionally, pathways related to insulin signaling, T2DM, p53, and cancer signaling were highlighted, indicating these miRNAs' potential role in these chronic diseases.

### 3.6 PBMC gene expression

There were no significant interactions between kawakawa tea and water intake for *IL‐2*, *AMPK*, *TNF‐α*, and *CD36* genes (Table 5). However, significant intervention and time‐point interactions were observed for *IL‐6* (*p* = .04), *PPAR‐γ* (*p* = .02), and *FAS* (*p* = .05). A time‐point change for *PPAR‐γ* (*p* = .03) and *IL‐8* (*p* = .01) was also observed.

**TABLE 5:** *p*‐Values obtained from a two‐way repeated measures ANOVA test of IL‐2, IL‐6, IL‐8, TNF‐α, PPAR‐γ, AMPK, and FAS gene expression.

	Two‐way ANOVA *p*‐Value		
	Intervention	Time‐point	Interaction
IL‐2	.07	.12	.52
IL‐6	.13	.15	.**04**
IL‐8	.24	.**01**	.41
CD36	.22	.11	.77
TNF‐α	.53	.42	.42
PPAR‐γ	.35	.**03**	.**02**
AMPK	.25	.16	.55
FAS	.07	.**05**	.**05**

*Note*: Intervention (kawakawa tea or water); time‐point (0, 60, 120 min); Values of significance (*p* ≤ .05) are presented in bold.

Abbreviations: AMPK, AMP‐activated protein kinase; CD36, cluster of differentiation 36; FAS, fas cell surface death receptor; IL‐2, interleukin‐2; IL‐6, interleukin‐6; IL‐8, interleukin‐8; PPAR‐γ, peroxisome proliferator‐activated receptor‐gamma; TNF‐α, tumor necrosis factor‐alpha.

**FIGURE 5:**
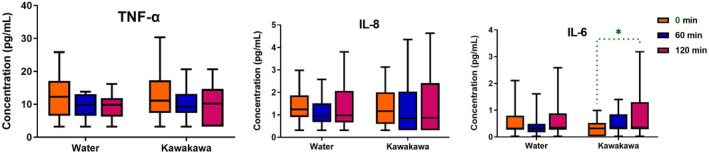
Comparison of inflammatory cytokines TNF‐α, IL‐8, and IL‐6 following kawakawa tea intake and water at fasting (0 min), 60 min, and 120 min. Different colors represent time points at fasting (0 min, orange color), at 60 min (Blue color), and at 120 min (Red color).; *p ≤ .05.

*IL‐8* and *IL‐6* demonstrated statistically significant changes in their relative gene expression between baseline and 120 min following kawakawa tea consumption. *PPAR‐γ* expression was significantly decreased at 60 min post kawakawa tea consumption. *CRP* was too lowly expressed to be identified in the current sample set (Figure 5).

### 3.7 Plasma cytokines

**FIGURE 6:**
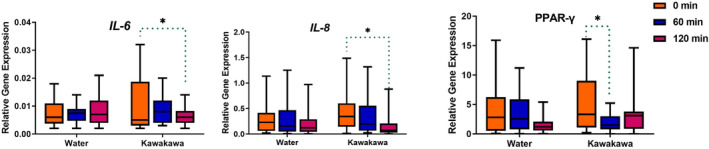
Relative gene expression of IL6, IL8, and PPAR‐γ following water and kawakawa tea intake at baseline (pre), 60, and 120 min. Different colors represent time points at fasting (0 min, orange color), at 60 min (Blue color), and at 120 min (Red color). *p ≤ .05.

Plasma levels of inflammatory cytokines before and after each intervention over the period of 120 min are shown in Figure 6. TNF‐α and IL‐8 levels were not different between the kawakawa intervention and the water control. However, IL‐6 showed a significant increase at 120 min in the kawakawa intervention compared to baseline. The abundance of both IL‐1β and IL‐2 were below the detectable threshold levels.

## 4 DISCUSSION

The present study aimed to investigate the effects of kawakawa tea (4 g dried leaves in 250 mL hot water) intake, compared to control (hot water) on the postprandial plasma abundances of miRNAs involved in metabolic‐related pathways and their respective gene and protein targets. As hypothesized, the current study observed a differential abundance of nine miRNAs (hsa‐miR‐16‐5p, ‐17‐5p, ‐21‐5p, ‐122‐5p, ‐144‐3p, ‐221‐3p, ‐223‐3p, ‐320a‐5p, and let‐7g‐5p), three mRNAs (*IL‐6, IL‐8* and *PPAR‐γ*), and a cytokine (IL‐6) following acute consumption of kawakawa tea prior to a high‐glycemic meal over the postprandial period of 120 min. Target gene prediction analysis revealed that these miRNAs play a role in altering the expression of genes involved in regulation of complex cardio‐metabolic diseases, as evidenced by their involvement in pathways such as insulin signaling, T2DM, p53, and cancer signaling. To our knowledge, the present study is the first time a human dietary intervention with kawakawa tea has provided insights into the mechanisms underlying its health effects.

Growing evidence has shown various diets to alter the abundance of plasma miRNA profiles that are involved in regulating important metabolic signaling pathways (Giardina et al., 2019; López de las Hazas et al., 1999; Ramzan et al., 2019). In particular, an association between the consumption of dietary phytochemicals and plasma miRNA levels has been observed, indicating dietary supplementation with bioactives could represent an effective strategy to modify the risk of chronic disease development (D'Amore et al., 2016; Marques‐Rocha et al., 2016; Tili et al., 2010; Tomé‐Carneiro et al., 2013). In this study, the immediate consumption of kawakawa tea before a high‐glycemic meal influenced the expression of nine miRNAs. These miRNAs have previously demonstrated dysregulated profiles in individuals with metabolic disorders such as prediabetes, T2DM, MetS, and obesity. Notably, hsa‐miR‐16‐5p, found at reduced levels in Brazilian women with MetS (Brandão‐Lima et al., 2022), has also shown a positive correlation with insulin sensitivity in non‐diabetic, weight‐stable individuals. Furthermore, hsa‐miR‐17‐5p, hsa‐miR‐21‐5p, and hsa‐miR‐320a are shown to have decreased abundance in metabolically dysregulated individuals compared to healthy counterparts (Zampetaki et al., 2010). The increased abundance of these miRNAs observed on intake of kawakawa tea indicates potential health benefits associated with acute kawakawa tea consumption.

While kawakawa tea intake was found to upregulate the abundance of 7 miRNAs compared to water, the study also observed a decrease in the abundance of two miRNAs (hsa‐miR‐221‐3p and hsa‐miR‐223‐3p). This is interesting as hsa‐miR‐221‐3p has increased plasma abundance in individuals with obesity, insulin resistance, and MetS. On the other hand, the decreased abundance of hsa‐miR‐223‐3p could be attributed to the decreased expression of *PPARγ. PPARγ* is identified as a direct regulator of this miRNA in adipose tissue (Chang et al., 2023). Given this study's findings of reduced abundance of both *PPARγ* and hsa‐miR‐223‐3p following kawakawa tea intake, it remains to be clarified whether kawakawa tea has a modulatory impact on inflammation and related pathways by influencing the hsa‐miR‐223‐3p/PPARγ axis.

A significant reduction in the expression of two pro‐inflammatory genes (*IL‐6* and *IL‐8*) after consuming kawakawa tea demonstrates potential anti‐inflammatory effects (Domingos et al., 2020). Unexpectedly, reduced gene expression did not correspond to changes in protein levels, defying the anticipated decrease in protein expression following decreased gene expression (Li & Xie, 2011). Despite this, an increase in IL‐6 cytokine levels was observed following kawakawa tea intakes. This rise in IL‐6 may be attributed to its pleiotropic nature, allowing it to exhibit both pro‐ and anti‐inflammatory functions. For example, during acute immune responses, IL‐6 can decrease pro‐inflammatory cytokines like TNF‐α without reducing the production of anti‐inflammatory cytokines such as IL‐1Ra (Gabay, 2006). Although not measured in our study, the consumption of kawakawa might impact the generation of pro‐inflammatory cytokines differently in various tissues, such as skeletal muscles, and PBMCs may not accurately represent these tissue‐specific alterations (Starkie et al., 2001a). For example, Starkie et al. (2001b) observed increased plasma IL‐6 concentrations immediately and at 2 h after prolonged running, suggesting skeletal muscle as a potential source of circulating IL‐6 rather than monocytes. This is corroborated by Ostrowski et al. (1998), who found *IL‐6* expression in the skeletal muscle but not in PBMCs of individuals engaged in prolonged running.

Although evidence strongly implicates hsa‐miR‐15a‐5p (Ramzan, D'Souza, Durainayagam, Milan, Roy, et al., 2020), hsa‐miR‐222‐3p (de Candia et al., 2017), and hsa‐miR‐21‐5p (La Sala et al., 2019) in insulin signaling pathways, this present study did not observe changes in their levels in response to kawakawa tea or water. Changes in miRNA expression due to dietary modifications depend on various factors, such as the duration of exposure, gender, and health status. For example, studies have shown that the impact of dietary interventions on miRNA expression profiles can vary based on how long an individual has been exposed to specific dietary changes. Additionally, the response may differ between genders, highlighting the need for gender‐specific considerations in understanding miRNA regulation through diet. Moreover, the health status of individuals plays a crucial role in shaping miRNA responses to dietary modulation. Individuals with different health conditions may exhibit distinct miRNA expression patterns in response to dietary changes. For instance, the miRNA expression profile in individuals with metabolic disorders like T2DM or obesity may differ from that in healthy individuals when subjected to the same dietary interventions.

The present study also did not observe any significant changes in the expression of the *CRP* gene throughout the postprandial period. Although CRP is considered an important marker of inflammation, it is usually expressed in response to chronic inflammatory‐related disorders such as diverse infections, rheumatoid arthritis, and CVD (Sproston & Ashworth, 2018). Since all the participants were self‐reported to be healthy, it is likely that CRP was not expressed due to an absence of prior inflammation in these individuals. It has also been shown that expression of CRP usually occurs within the initial 4–24 h following stimuli (Zwickey & Thompson, 2020). Owing that for this study, gene expression was measured at 60 and 120 min, it's conceivable that *CRP* might not have been expressed within this short duration. However, it's crucial to note that these observations remain associative with no clear mechanistic understanding of how kawakawa influences *CRP* and inflammation.

### 4.1 Limitations and further research

The consumption of kawakawa presents promising potential for anti‐inflammatory properties through the modulation of gene and miRNA expressions. However, it's crucial to acknowledge several limitations in this current study. Sexual dimorphism and ethnicity significantly affect the abundance of many miRNAs in the plasma (Guo et al., 2017; Huang et al., 2011). However, this study was not powered enough to understand the sexual dimorphism of these changes. This initial human dietary intervention trial with kawakawa, conducted as a pilot with healthy participants, establishes fundamental insights for shaping future research questions. To improve the generalizability and translatability of these findings, forthcoming investigations should involve individuals with diverse metabolic statuses. This study also undertook a limited and targeted qPCR‐based analysis of both miRNA and mRNA, with the latter performed only in circulatory PBMCs. Although these cells have been widely used as proxy tissue to understand whole‐body metabolic status, they are not always appropriate surrogates (Hedges et al., 2019). Lastly, there isn't a universally agreed‐upon minimal threshold for profiling plasma miRNA abundance (Witwer & Halushka, 2016). This lack of consensus underscores that plasma miRNA abundance may not consistently serve as an appropriate biomarker when distinguishing between biological significance and experimental noise. Thus, interpreting the biological significance of minor changes, as seen in the current study, remains challenging. Consequently, integrating high‐throughput small RNA sequencing strategies and larger population cohorts in subsequent analysis would provide a more comprehensive evaluation of the biological significance of the global regulation of non‐coding and coding RNA transcripts.

## 5 CONCLUSION

This study demonstrated that consuming kawakawa tea before a high‐glycemic meal influenced the circulatory levels of specific miRNAs. The target genes of these miRNAs primarily participate in pathways related to inflammation and insulin signaling. These findings contribute to advancing our understanding of the molecular mechanisms underlying the positive health effects associated with kawakawa tea consumption.

## AUTHOR CONTRIBUTIONS

FR, MF, JM‐C, CP, and RM designed TOAST. FR, RJ, and PS conducted and coordinated the dietary intervention study. ST and JG conducted the laboratory experiments. ST and JG conducted the statistical analysis. FR wrote the paper. All authors provided content and feedback on the manuscript. FR has primary responsibility for the final content of the manuscript.

## FUNDING INFORMATION

This study was financially supported by Wakatū Incorporation, Liggins Institute, University of Auckland, and the New Zealand National Science Challenge, High‐Value Nutrition Program.

## CONFLICT OF INTEREST STATEMENT

The authors declare no conflict of interest.

## Data Availability

The data generated and analyzed during this study are available from the corresponding author upon reasonable request.

## References

[fsn34450-bib-0001] Åkerman, L. , Casas, R. , Ludvigsson, J. , Tavira, B. , & Skoglund, C. (2018). Serum miRNA levels are related to glucose homeostasis and islet autoantibodies in children with high risk for type 1 diabetes. PLoS One, 13, e0191067. 29346396 10.1371/journal.pone.0191067 PMC5773164

[fsn34450-bib-0002] Arenas, C. , Toma, C. , Cormand, B. , & Irigoien, I. (2016). Identifying extreme observations, outliers and noise in clinical and genetic data. Current Bioinformatics, 12, 101–117.

[fsn34450-bib-0003] Brandão‐Lima, P. N. , de Carvalho, G. B. , Payolla, T. B. , Sarti, F. M. , Fisberg, R. M. , Malcomson, F. C. , Mathers, J. C. , & Rogero, M. M. (2022). Circulating microRNAs showed specific responses according to metabolic syndrome components and sex of adults from a population‐based study. Metabolites, 13. 10.3390/METABO13010002 PMC9861536 36676927

[fsn34450-bib-0004] Briggs, L. H. (1941). The essential oil of macropiper excelsum (kawakawa). Journal of the Society of Chemical Industry, 60, 210–212.

[fsn34450-bib-0005] Butts, C. A. , van Klink, J. W. , Joyce, N. I. , Paturi, G. , Hedderley, D. I. , Martell, S. , & Harvey, D. (2019). Composition and safety evaluation of tea from New Zealand kawakawa (*Piper excelsum*). Journal of Ethnopharmacology, 232, 110–118. 30572092 10.1016/j.jep.2018.12.029

[fsn34450-bib-0006] Chang, R. C. , Joloya, E. M. , Li, Z. , Shoucri, B. M. , Shioda, T. , & Blumberg, B. (2023). miR‐223 Plays a key role in obesogen‐enhanced adipogenesis in mesenchymal stem cells and in transgenerational obesity. Endocrinology, 164(5), bqad027. 36740725 10.1210/endocr/bqad027 PMC10282922

[fsn34450-bib-0007] Damanti, C. C. , Gaffo, E. , Lovisa, F. , Garbin, A. , Di Battista, P. , Gallingani, I. , Tosato, A. , Pillon, M. , Carraro, E. , Mascarin, M. , Elia, C. , Biffi, A. , Bortoluzzi, S. , & Mussolin, L. (2021). MiR‐26a‐5p as a reference to normalize MicroRNA qRT‐PCR levels in plasma exosomes of pediatric hematological malignancies. Cells, 10, 1. 10.3390/cells10010101 PMC7827902 33429910

[fsn34450-bib-0008] D'Amore, S. , Vacca, M. , Cariello, M. , Graziano, G. , D'Orazio, A. , Salvia, R. , Sasso, R. C. , Sabbà, C. , Palasciano, G. , & Moschetta, A. (2016). Genes and miRNA expression signatures in peripheral blood mononuclear cells in healthy subjects and patients with metabolic syndrome after acute intake of extra virgin olive oil. Biochimica et Biophysica Acta ‐ Molecular and Cell Biology of Lipids, 1861, 1671–1680. 10.1016/j.bbalip.2016.07.003 27422371

[fsn34450-bib-0009] de Candia, P. , Spinetti, G. , Specchia, C. , Sangalli, E. , La Sala, L. , Uccellatore, A. , Lupini, S. , Genovese, S. , Matarese, G. , & Ceriello, A. (2017). A unique plasma microRNA profile defines type 2 diabetes progression. PLoS One, 12, e0188980. 29200427 10.1371/journal.pone.0188980 PMC5714331

[fsn34450-bib-0010] Domingos, I. F. , Pereira‐Martins, D. A. , Sobreira, M. J. V. C. , Oliveira, R. T. D. , Alagbe, A. E. , Lanaro, C. , Albuquerque, D. M. , Blotta, M. H. S. L. , Araujo, A. S. , Costa, F. F. , Lucena‐Araujo, A. R. , Sonati, M. F. , Bezerra, M. A. C. , & Santos, M. N. N. (2020). High levels of proinflammatory cytokines IL‐6 and IL‐8 are associated with a poor clinical outcome in sickle cell anemia. Annals of Hematology, 99, 947–953. 32140892 10.1007/s00277-020-03978-8

[fsn34450-bib-0011] Eisenberg, E. , & Levanon, E. Y. (2013). Human housekeeping genes, revisited. Trends in Genetics, 29, 569–574. 23810203 10.1016/j.tig.2013.05.010

[fsn34450-bib-0012] El‐Khoury, V. , Pierson, S. , Kaoma, T. , Bernardin, F. , & Berchem, G. (2016). Assessing cellular and circulating miRNA recovery: The impact of the RNA isolation method and the quantity of input material. Scientific Reports, 6, 19529. 26787294 10.1038/srep19529 PMC4726450

[fsn34450-bib-0013] Elshelmani, H. , Brennan, I. , Kelly, D. J. , & Keegan, D. (2021). Differential circulating microRNA expression in age‐related macular degeneration. International Journal of Molecular Sciences, 22, 12321. 10.3390/IJMS222212321 34830203 PMC8625913

[fsn34450-bib-0014] Fan, Y. , Siklenka, K. , Arora, S. K. , Ribeiro, P. , Kimmins, S. , & Xia, J. (2016). miRNet – Dissecting miRNA‐target interactions and functional associations through network‐based visual analysis. Nucleic Acids Research, 44, W135–W141. 27105848 10.1093/nar/gkw288 PMC4987881

[fsn34450-bib-0015] Fatima, F. , & Nawaz, M. (2017). Long distance metabolic regulation through adipose‐derived circulating Exosomal miRNAs: A trail for RNA‐based therapies? Frontiers in Physiology, 8, 545. 28824444 10.3389/fphys.2017.00545 PMC5539684

[fsn34450-bib-0016] Gabay, C. (2006). Interleukin‐6 and chronic inflammation. Arthritis Research & Therapy, 8, S3. 10.1186/ar1917 PMC3226076 16899107

[fsn34450-bib-0017] Giardina, S. , Hernández‐Alonso, P. , Díaz‐López, A. , Salas‐Huetos, A. , Salas‐Salvadó, J. , & Bulló, M. (2019). Changes in circulating miRNAs in healthy overweight and obese subjects: Effect of diet composition and weight loss. Clinical Nutrition, 38, 438–443. 29233588 10.1016/j.clnu.2017.11.014

[fsn34450-bib-0018] Gong, R. , Lv, X. , & Liu, F. (2018). MiRNA‐17 encoded by the miR‐17‐92 cluster increases the potential for steatosis in hepatoma cells by targeting CYP7A1. Cellular & Molecular Biology Letters, 23, 16. 29721023 10.1186/s11658-018-0083-3 PMC5907481

[fsn34450-bib-0019] Guo, L. , Zhang, Q. , Ma, X. , Wang, J. , & Liang, T. (2017). miRNA and mRNA expression analysis reveals potential sex‐biased miRNA expression. Nature, 7, 39812. 10.1038/srep39812 PMC5206641 28045090

[fsn34450-bib-0020] Hedges, C. P. , Woodhead, J. S. T. , Wang, H. W. , Mitchell, C. J. , Cameron‐Smith, D. , Hickey, A. J. R. , & Merry, T. L. (2019). Peripheral blood mononuclear cells do not reflect skeletal muscle mitochondrial function or adaptation to high‐intensity interval training in healthy young men. Journal of Applied Physiology, 126, 454–461. 30571281 10.1152/japplphysiol.00777.2018

[fsn34450-bib-0021] Huang, R. S. , Gamazon, E. R. , Ziliak, D. , Wen, Y. , Im, H. K. , Zhang, W. , Wing, C. , Duan, S. , Bleibel, W. K. , Cox, N. J. , & Dolan, M. E. (2011). Population differences in microRNA expression and biological implications. RNA Biology, 8, 692–701. 21691150 10.4161/rna.8.4.16029 PMC3225983

[fsn34450-bib-0022] Jayaprakash, R. , Ramzan, F. , Miles‐Chan, J. L. , Foster, M. , Mithen, R. F. , & Pook, C. (2022). Exploring the chemical space of kawakawa leaf (*Piper excelsum*). Nutrients, 14, 5168. 36501198 10.3390/nu14235168 PMC9741024

[fsn34450-bib-0023] Kaplon, J. , van Dam, L. , & Peeper, D. (2022). Two‐way communication between the metabolic and cell cycle machineries: the molecular basis. Cell Cycle, 2015, 14. 10.1080/15384101.2015.1044172 PMC4615069 26038996

[fsn34450-bib-0024] Koia, J. H. , & Shepherd, P. (2020). The potential of anti‐diabetic Rākau Rongoā (Māori herbal medicine) to treat type 2 diabetes mellitus (T2DM) mate Huka: A review. Frontiers in Pharmacology, 11, 935. 32694996 10.3389/fphar.2020.00935 PMC7339977

[fsn34450-bib-0025] Končarević, S. , Lößner, C. , Kuhn, K. , Prinz, T. , Pike, I. , & Zucht, H.‐D. (2014). In‐depth profiling of the peripheral blood mononuclear cells proteome for clinical blood proteomics. Int J Proteomics, 2014, 129259. 24724028 10.1155/2014/129259 PMC3958665

[fsn34450-bib-0026] La Sala, L. , Mrakic‐Sposta, S. , Tagliabue, E. , Prattichizzo, F. , Micheloni, S. , Sangalli, E. , Specchia, C. , Uccellatore, A. C. , Lupini, S. , Spinetti, G. , De Candia, P. , & Ceriello, A. (2019). Circulating microRNA‐21 is an early predictor of ROS‐mediated damage in subjects with high risk of developing diabetes and in drug‐naïve T2D. Cardiovascular Diabetology, 18, 1. 30803440 10.1186/s12933-019-0824-2 PMC6388471

[fsn34450-bib-1004] Lee, J. Y. , & Park, W. (2011). Anti‐inflammatory effect of myristicin on RAW 264.7 macrophages stimulated with polyinosinic‐polycytidylic acid. Molecules, 16(8), 7132–7142. 21991618 10.3390/molecules16087132 PMC6264243

[fsn34450-bib-0027] Lei, J. , Burgess, E. J. , Richardson, A. T. B. , Hawkins, B. C. , Baird, S. K. , Smallfield, B. M. , Van Klink, J. W. , & Perry, N. B. (2015). Cytotoxic amides from fruits of kawakawa, macropiper excelsum. Planta Medica, 81, 1163–1168. 26039266 10.1055/s-0035-1546106

[fsn34450-bib-0028] Li, G. W. , & Xie, X. S. (2011). Central dogma at the single‐molecule level in living cells. Nature, 475, 308–315. 21776076 10.1038/nature10315 PMC3600414

[fsn34450-bib-0030] López de las Hazas, M. C. , Gil‐Zamorano, J. , Cofán, M. , Mantilla‐Escalante, D. C. , Garcia‐Ruiz, A. , del Pozo‐Acebo, L. , Pastor, O. , Yañez‐Mo, M. , Mazzeo, C. , Serra‐Mir, M. , Doménech, M. , Valls‐Pedret, C. , Rajaram, S. , Sabaté, J. , Ros, E. , Sala‐Vila, A. , & Dávalos, A. (1999). One‐year dietary supplementation with walnuts modifies exosomal miRNA in elderly subjects. European Journal of Nutrition, 2021, 60–2011. 10.1007/s00394-020-02390-2 32979076

[fsn34450-bib-0031] Marques‐Rocha, J. L. , Milagro, F. I. , Mansego, M. L. , Zulet, M. A. , Bressan, J. , & Martínez, J. A. (2016). Expression of inflammation‐related miRNAs in white blood cells from subjects with metabolic syndrome after 8 wk of following a Mediterranean diet–based weight loss program. Nutrition, 32, 48–55. 26421388 10.1016/j.nut.2015.06.008

[fsn34450-bib-0032] Max, K. E. A. , Bertram, K. , Akat, K. M. , Bogardus, K. A. , Li, J. , Morozov, P. , Ben‐Dov, I. Z. , Li, X. , Weiss, Z. R. , Azizian, A. , Sopeyin, A. , Diacovo, T. G. , Adamidi, C. , Williams, Z. , & Tuschl, T. (2018). Human plasma and serum extracellular small RNA reference profiles and their clinical utility. Proceedings of the National Academy of Sciences of the United States of America, 115, E5334. 29777089 10.1073/pnas.1714397115 PMC6003356

[fsn34450-bib-0033] Metallo, C. M. , & Vander Heiden, M. G. (2013). Understanding metabolic regulation and its influence on cell physiology. Molecular Cell, 49, 388–398. 23395269 10.1016/j.molcel.2013.01.018 PMC3569837

[fsn34450-bib-0034] O'Brien, J. , Hayder, H. , Zayed, Y. , & Peng, C. (2018). Overview of MicroRNA biogenesis, mechanisms of actions, and circulation. Frontiers in Endocrinology (Lausanne), 9, 402. 10.3389/fendo.2018.00402 PMC6085463 30123182

[fsn34450-bib-0035] Ostrowski, K. , Rohde, T. , Zacho, M. , Asp, S. , & Pedersen, B. K. (1998). Evidence that interleukin‐6 is produced in human skeletal muscle during prolonged running. The Journal of Physiology, 508, 949–953. 9518745 10.1111/j.1469-7793.1998.949bp.x PMC2230908

[fsn34450-bib-0036] Ramzan, F. , D'Souza, R. F. , Durainayagam, B. R. , Milan, A. M. , Markworth, J. F. , Miranda‐Soberanis, V. , Sequeira, I. R. , Roy, N. C. , Poppitt, S. D. , Mitchell, C. J. , & Cameron‐Smith, D. (2020). Circulatory miRNA biomarkers of metabolic syndrome. Acta Diabetologica, 57, 203–214. 31435783 10.1007/s00592-019-01406-6

[fsn34450-bib-0037] Ramzan, F. , D'Souza, R. F. , Durainayagam, B. R. , Milan, A. M. , Roy, N. C. , Kruger, M. C. , Henry, C. J. , Mitchell, C. J. , & Cameron‐Smith, D. (2020). Inflexibility of the plasma miRNA response following a high‐carbohydrate meal in overweight insulin‐resistant women. Genes & Nutrition, 15, 1. 32042348 10.1186/s12263-020-0660-8 PMC7001289

[fsn34450-bib-0038] Ramzan, F. , Jayaprakash, R. , Pook, C. , Foster, M. , Miles‐Chan, J. L. , & Mithen, R. (2022). Acute effects of kawakawa (*Piper excelsum*) intake on postprandial glycemic and insulinaemic response in a healthy population. Nutrients, 14, 1638. 35458200 10.3390/nu14081638 PMC9032225

[fsn34450-bib-0039] Ramzan, F. , Mitchell, C. J. , Milan, A. M. , Schierding, W. , Zeng, N. , Sharma, P. , Mitchell, S. M. , D'Souza, R. F. , Knowles, S. O. , Roy, N. C. , Sjödin, A. , Wagner, K. H. , & Cameron‐Smith, D. (2019). Comprehensive profiling of the circulatory miRNAome response to a high protein diet in elderly men: A potential role in inflammatory response modulation. Molecular Nutrition & Food Research, 63, 1800811. 10.1002/mnfr.201800811 30892810

[fsn34450-bib-0040] Salgado, A. L. F. D. A. , de Carvalho, L. , Oliveira, A. C. , dos Santos, V. N. , Vieira, J. G. , & Parise, E. R. (2010). Insulin resistance index (HOMA‐IR) in the differentiation of patients with non‐alcoholic fatty liver disease and healthy individuals. Arquivos de Gastroenterologia, 47, 165–169. 20721461 10.1590/s0004-28032010000200009

[fsn34450-bib-0041] Schmittgen, T. D. , & Livak, K. J. (2008). Analyzing real‐time PCR data by the comparative CT method. Nature Protocols, 3, 1101–1108. 18546601 10.1038/nprot.2008.73

[fsn34450-bib-0042] Sepulveda, J. L. (2019). Accurate results in the clinical laboratory: A guide to error detection and correction (2nd ed., p. 101). Elsevier.

[fsn34450-bib-0043] Shah, J. S. , Soon, P. S. , & Marsh, D. J. (2016). Comparison of methodologies to detect low levels of hemolysis in serum for accurate assessment of serum microRNAs. PLoS One, 11, e0153200. 27054342 10.1371/journal.pone.0153200 PMC4824492

[fsn34450-bib-0044] Shuai Jiang, W. Y. (2016). Current view of microRNA processing. Signal Transduction Insights, 5, 9.

[fsn34450-bib-0045] Slack, C. (2017). Ras signaling in aging and metabolic regulation. Nutrition and Healthy Aging, 4, 195–205. 29276789 10.3233/NHA-160021 PMC5734121

[fsn34450-bib-0046] Sproston, N. R. , & Ashworth, J. J. (2018). Role of C‐reactive protein at sites of inflammation and infection. Frontiers in Immunology, 9, 1. 29706967 10.3389/fimmu.2018.00754 PMC5908901

[fsn34450-bib-0047] Starkie, R. L. , Rolland, J. , Angus, D. J. , Anderson, M. J. , & Febbraio, M. A. (2001a). Circulating monocytes are not the source of elevations in plasma IL‐6 and TNF‐α levels after prolonged running. American Journal of Physiology. Cell Physiology, 280, 769–C774. 10.1152/ajpcell.2001.280.4.C769 11245592

[fsn34450-bib-0048] Tili, E. , Michaille, J. J. , Adair, B. , Alder, H. , Limagne, E. , Taccioli, C. , Ferracin, M. , Delmas, D. , Latruffe, N. , & Croce, C. M. (2010). Resveratrol decreases the levels of miR‐155 by upregulating miR‐663, a microRNA targeting JunB and JunD. Carcinogenesis, 31, 1561–1566. 20622002 10.1093/carcin/bgq143 PMC4647642

[fsn34450-bib-0049] Tomé‐Carneiro, J. , Larrosa, M. , Yáñez‐Gascón, M. J. , Dávalos, A. , Gil‐Zamorano, J. , Gonzálvez, M. , García‐Almagro, F. J. , Ruiz Ros, J. A. , Tomás‐Barberán, F. A. , Espín, J. C. , & García‐Conesa, M. T. (2013). One‐year supplementation with a grape extract containing resveratrol modulates inflammatory‐related microRNAs and cytokines expression in peripheral blood mononuclear cells of type 2 diabetes and hypertensive patients with coronary artery disease. Pharmacological Research, 72, 69–82. 23557933 10.1016/j.phrs.2013.03.011

[fsn34450-bib-0050] van de Moosdijk, A. A. A. , & van Amerongen, R. (2016). Identification of reliable reference genes for qRT‐PCR studies of the developing mouse mammary gland. Scientific Reports, 6, 35595. 27752147 10.1038/srep35595 PMC5067587

[fsn34450-bib-0051] Vandesompele, J. , de Preter, K. , Pattyn, F. , Poppe, B. , van Roy, N. , de Paepe, A. , & Speleman, F. (2002a). Accurate normalization of real‐time quantitative RT‐PCR data by geometric averaging of multiple internal control genes. Genome Biology, 3, 1. 10.1186/gb-2002-3-7-research0034 PMC126239 12184808

[fsn34450-bib-0052] Wang, J. , Zhu, Y. , Jin, F. , Tang, L. , He, Z. , & He, Z. (2016). Differential expression of circulating microRNAs in blood and haematoma samples from patients with intracerebral haemorrhage. The Journal of International Medical Research, 44, 419–432. 27020596 10.1177/0300060516630852 PMC5536709

[fsn34450-bib-0053] Witwer, K. W. , & Halushka, M. K. (2016). Toward the promise of microRNAs – Enhancing reproducibility and rigor in microRNA research. RNA Biology, 13, 1103–1116. 27645402 10.1080/15476286.2016.1236172 PMC5100345

[fsn34450-bib-0055] Ye, J. , Coulouris, G. , Zaretskaya, I. , Cutcutache, I. , Rozen, S. , & Madden, T. L. (2012). Primer‐BLAST: A tool to design target‐specific primers for polymerase chain reaction. BMC Bioinformatics, 13, 134. 22708584 10.1186/1471-2105-13-134 PMC3412702

[fsn34450-bib-0056] Zampetaki, A. , Kiechl, S. , Drozdov, I. , Willeit, P. , Mayr, U. , Prokopi, M. , Mayr, A. , Weger, S. , Oberhollenzer, F. , Bonora, E. , Shah, A. , Willeit, J. , & Mayr, M. (2010). Plasma microRNA profiling reveals loss of endothelial MiR‐126 and other microRNAs in type 2 diabetes. Circulation Research, 107, 810–817. 20651284 10.1161/CIRCRESAHA.110.226357

[fsn34450-bib-0057] Zwickey, H. , & Thompson, B. (2020). Immune function assessment. In Textbook of natural medicine (p. 157). Churchill Livingstone.

